# Enzymatic Synthesis of Biologically Active *H*-Phosphinic Analogue of α-Ketoglutarate

**DOI:** 10.3390/biom14121574

**Published:** 2024-12-10

**Authors:** Vsevolod L. Filonov, Maxim A. Khomutov, Yaroslav V. Tkachev, Artem V. Udod, Dmitry V. Yanvarev, Fabio Giovannercole, Elena N. Khurs, Sergei N. Kochetkov, Daniela De Biase, Alex R. Khomutov

**Affiliations:** 1 Engelhardt Institute of Molecular Biology, Russian Academy of Sciences, Vavilov St., 32, 119991 Moscow, Russia; filonov_vsevolod@mail.ru (V.L.F.); makhomutov@mail.ru (M.A.K.); yaroslav@eimb.ru (Y.V.T.); art.udod@live.com (A.V.U.); yanvarev@eimb.ru (D.V.Y.); enkhurs@yandex.ru (E.N.K.); snk1952@gmail.com (S.N.K.); 2 Département de Biologie, Université de Namur, Rue de Bruxelles 61, 5000 Namur, Belgium; fabio.giovannercole@gmail.com; 3 Department of Medico-Surgical Sciences and Biotechnologies, Sapienza University of Rome, Corso della Repubblica 79, 04100 Latina, Italy; daniela.debiase@uniroma1.it

**Keywords:** *H*-phosphinic analogues of glutamate, *H*-phosphinic analogue of α-ketoglutarate, glutamate dehydrogenase, glutamate metabolism

## Abstract

Amino acid analogues with a phosphorus-containing moiety replacing the carboxylic group are promising sources of biologically active compounds. The *H*-phosphinic group, with hydrogen–phosphorus–carbon (H-P-C) bonds and a flattened tetrahedral configuration, is a bioisostere of the carboxylic group. Consequently, amino-*H*-phosphinic acids undergo substrate-like enzymatic transformations, leading to new biologically active metabolites. Previous studies employing NMR-based metabolomic and proteomic analyses show that in *Escherichia coli*, α-KG-γ-P_H_ (the distal *H*-phosphinic analogue of α-ketoglutarate) can be converted into *L*-Glu-γ-P_H_. Notably, α-KG-γ-P_H_ and *L*-Glu-γ-P_H_ are antibacterial compounds, but their intracellular targets only partially overlap. *L*-Glu-γ-P_H_ is known to be a substrate of aspartate transaminase and glutamate decarboxylase, but its substrate properties with NAD^+^-dependent glutamate dehydrogenase (GDH) have never been investigated. Compounds containing *P-H* bonds are strong reducing agents; therefore, enzymatic NAD^+^-dependent oxidation is not self-evident. Herein, we demonstrate that *L*-Glu-γ-P_H_ is a substrate of eukaryotic GDH and that the pH optimum of *L*-Glu-γ-P_H_ NAD^+^-dependent oxidative deamination is shifted to a slightly alkaline pH range compared to *L*-glutamate. By ^31^P NMR, we observe that α-KG-γ-P_H_ exists in a pH-dependent equilibrium of keto and germinal diol forms. Furthermore, the stereospecific enzymatic synthesis of α-KG-γ-P_H_ from *L*-Glu-γ-P_H_ using GDH is a possible route for its bio-based synthesis.

## 1. Introduction

**Figure 1:**
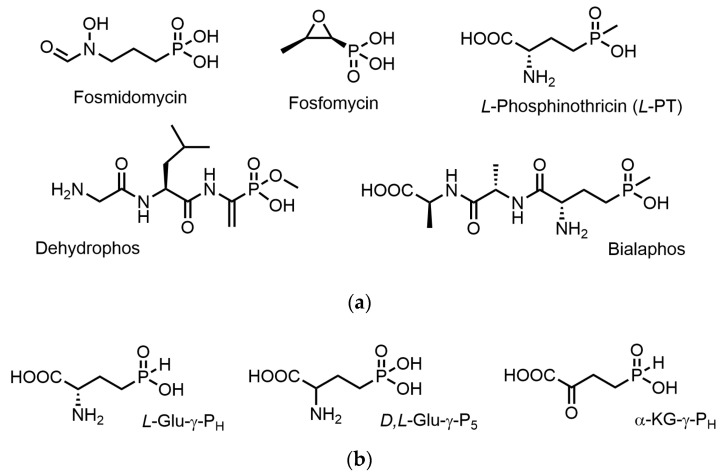
Chemical structure of the compounds containing phosphonic and phosphinic groups. (**a**) Well-known phosphorus-containing compounds with pharmacological or herbicidal activity. (**b**) Compounds of interest in this study, i.e., *H*-phosphinic analogues of *L*-glutamate and α-ketoglutarate (*L*-Glu-γ-P_H_ and α-KG-γ-P_H_), respectively, and phosphonic analogue of glutamate (*D,L*-Glu-γ-P_5_).

Secondary metabolites synthesized by microorganisms and plants are a limitless source of biologically active compounds, and nearly two-thirds of all human medicines are derived from these natural compounds [1], many of which express antimicrobial activities [2,3]. Specifically, microorganisms synthesize a plethora of different compounds with biochemically stable carbon–phosphorus (P-C) bonds. Examples of antibacterials with such bonds (Figure 1a) include fosfomycin (an inhibitor of UDP-N-acetylglucosamine enolpyruvyl transferase [4]) and fosmidomycin (an inhibitor of 1-deoxy-*D*-xylulose-5-phosphate reductoisomerase [5]); the latter is active against different enterobacteria [6] and parasites of the *Plasmodium* genus as well [5].

Aminophosphonic acids with a -P(O)(OH)_2_ group or aminophosphinic acids with a -P(O)(CH_3_)OH group poorly penetrate cells; therefore, properly designed transport forms are needed to deliver these compounds. That is why many biologically active compounds of this type exist in nature in the form of short phosphorus-containing peptides, which are non-toxic for the producing microorganism. These peptides can be taken up by other microorganisms via peptidyl permeases and then cleaved within the cell by peptidases, releasing the corresponding phosphorus-containing amino acid analogues [7]. A prodrug of this type is the tripeptide Bialaphos (Figure 1a), which is a widely used herbicide. Bialaphos effectively penetrates cells, and once cleaved, it releases *L*-phosphinothricin (*L*-PT; Figure 1a), one of the most powerful inhibitors of glutamine synthetase [8]. Dehydrophos (Figure 1a) is an example of a “double prodrug” because once taken up by bacteria using peptidyl permeases, it is cleaved by peptidases and releases the phosphorus-containing analogue of dehydroalanine that spontaneously rearranges to the methyl ester of the phosphonic analogue of pyruvate [9], which is a powerful inhibitor of pyruvate dehydrogenase [10,11].

The use of “genome mining” may revolutionize the discovery of novel biologically active compounds, including natural products with P-C bonds, whose properties make them excellent objects for large-scale “genome mining” [3,12]. This is because most of the compounds with P-C bonds are derived from phosphoenolpyruvate, which is isomerized to phosphonopyruvate by phosphoenolpyruvate mutase and coded by a gene, which represents a convenient marker for “genome mining”. The subsequent decarboxylation of phosphonopyruvate leads to phosphonoacetaldehyde [2,12], which, either in this form or after some transformations, serves as a precursor for the phosphonates of different classes. The analysis of genome databases allowed authors to conclude that up to 10–15% of bacteria have genes of the two aforementioned enzymes and are capable of producing phosphonates. Notably, through “genome mining” in 10,000 actinomycetes, not only well-known compounds with P-C bonds were confirmed, but also 19 new substances were discovered, including some with antibacterial activity [12].

A rational approach to designing novel biologically active compounds starts with selecting a metabolic target. In this work, the metabolism of glutamic acid was chosen despite its diversity and high content of glutamate in bacteria. Glutamate concentration in *E. coli* is 100 and 150 mM when the carbon source in the growth medium is glucose or glycerol, respectively [13]. One of our lead compounds is 2-amino-4-(*H*-phosphinoyl)butyric acid (desmethylphosphinithricin; hereafter, *L*-Glu-γ-P_H_; Figure 1b), the phosphorus-containing analogue of glutamic acid, the distal carboxyl group of which is substituted with a *H*-phosphinic moiety. *L*-Glu-γ-P_H_ was first isolated from *Streptomyces hygroscopicus* and *S. viridochromogenes* [14] as one of the key intermediates in the herbicide Bialaphos’ (Figure 1a) biosynthesis [15]. In a mutant form of *S. hygroscopicus* with the blocked Bialaphos biosynthetic pathway, *L*-Glu-γ-P_H_ was shown to accumulate and inhibit the growth of this microorganism at 10 µg/mL [16]. This is why in the course of the biosynthesis of Bialaphos, *L*-Glu-γ-P_H_ is initially acetylated to make the compound inactive and protect *Streptomyces* from the action of this antibiotic. As a matter of fact, the removal of the acetyl group takes place at one of the last steps of Bialaphos biosynthesis [16]. In addition, *L*-Glu-γ-P_H_ was also found in *Nonomureae* sp. NRRL B-24552 [12]. We have demonstrated that *L*-Glu-γ-P_H_ inhibits the growth of the *E. coli* K12 MG1655 strain [17] and, more recently, have provided evidence that the Minimal Inhibiting Concentration (MIC) of *L*-Glu-γ-P_H_ may be significantly lowered if *L*-Glu-γ-P_H_ is converted into the dipeptide *L*-Leu-*L*-Glu-γ-P_H_, which very likely improves penetration in the bacteria cells via the peptidyl permease system and, upon cleavage by peptidases, increases *L*-Glu-γ-P_H_ intracellular concentration [18]. 

Considering the ease of interconversion of glutamic and α-ketoglutaric acids, the use of α-ketoglutarate derivatives as prodrugs is also of particular interest. Recent data clearly showed that the MIC of 2-oxo-4-(*H*-phosphinoyl)butyric acid (hereafter, α-KG-γ-P_H_; Figure 1b) on *E. coli* was in the same µM range as *L*-Glu-γ-P_H_ [19]. A notable finding was that *E. coli* converted α-KG-γ-P_H_ into *L*-Glu-γ-P_H_, whereas the opposite was not observed [19]. Moreover, NMR-based metabolomic and proteomic analyses have shown that the overall effect of α-KG-γ-P_H_ on *E. coli* is more marked than the effect of *L*-Glu-γ-P_H_ [19]. 

Glutamate dehydrogenase (GDH) catalyzes the reversible oxidative deamination of *L*-glutamate, yielding α-ketoglutarate. This transformation is one of the most important pathways of nitrogen metabolism since it affects both amino acid metabolism and the tricarboxylic acid cycle, as well as being involved in maintaining the acid-base and redox balance of the cell [20]. However, the possibility of NAD^+^-dependent enzymatic oxidative deamination of *L*-Glu-γ-P_H_ to yield α-KG-γ-P_H_ is not self-evident. Moreover, the recent metabolomic data suggest that the reverse reaction is more likely to occur, at least in *E. coli* [19].

Herein, we describe for the first time that starting from *L*-Glu-γ-P_H_, the enzymatic synthesis of α-KG-γ-P_H_ with the use of NAD^+^-dependent bovine liver GDH is feasible and that this can be exploited to synthesize preparative amounts of α-KG-γ-P_H_. In addition, insights into the interaction of *L*-Glu-γ-P_H_ and related aminophosphonates (*D*,*L*-Glu-γ-P_5_ and *D,L*-PT, Figure 1a,b) with GDH are presented.

## 2. Materials and Methods

### 2.1. Materials

*rac*-Glu-γ-P_H_, *L*-Glu-γ-P_H_ and *D*-Glu-γ-P_H_ were synthesized, as described in [17]; *rac*-Glu-γ-P_5_ was purchased from Santa Cruz Biotechnology (Dallas, TX, USA); and *rac*-PT (ammonium glufosinate), *L*-glutamate, Tris base, NAD^+^, *L*-leucine, 37% aq HCl, NaOH, trifluoroacetic acid and HPLC grade acetonitrile were purchased from Sigma (St. Louis, MO, USA).

Bovine liver type II *L*-Glutamic Dehydrogenase, Cat. No. G2626 (a 50% glycerol solution, ≥35 U/mg protein calculated using the definition that one GDH unit reduces “1.0 micromole of α-ketoglutarate to *L*-glutamate per minute at pH 7.3 at 25 °C, in the presence of ammonium ions”) was purchased from Sigma (USA). The enzyme activity was assayed, essentially as described in [21], but in 100 mM Tris–HCl buffer at the indicated pH values. 

Ion-exchange chromatography was carried out on a Dowex 50W X8 (H^+^ form, 100–200 mesh; BioRad, Hercules, CA, USA). HPLC was carried out on a semi-preparative reverse-phase column 250 × 10 mm ReproSil-Pur C18-AQ (5 µm, Dr. Maisch GmbH, Ammerbuch, Germany). HPLC was performed on a Gilson chromatographic system (based on 305 and 302 pumps) equipped with a UV/VIS-151 detector and monitored by an AD-24 controller (Ampersand Ltd., Moscow, Russia) using the software Multichrom v. 3.4 (Ampersand Ltd., Russia). The elution conditions are specified in the text.

^1^H, ^13^C and ^31^P NMR spectra were acquired at 303 K, using a 300 MHz Avance III spectrometer (Bruker, Billerica, MA, USA) in D_2_O or H_2_O/10% D_2_O solution. In the latter case, the water signal was suppressed using an “excitation sculpting” pulse sequence [22]. The calibration of ^1^H spectra was performed using sodium 3-trimethyl-1-propanesulfonate (DSS) as the internal standard, and ^31^P—using the frequency ratio *Ξ* = 40.480742% of 85% H_3_PO_4_, as recommended by the IUPAC [23]. In the exchange–correlation experiments (^31^P-^31^P NOESY), a mixing time of 4 s was used.

### 2.2. Enzymatic Activity Assay and Kinetic Parameter Calculation

Reaction mixtures (500 μL) containing either *L*-glutamate (10 mM), *rac*-Glu-γ-P_H_ (10 mM) or *L*-Glu-γ-P_H_ (10 mM) included NAD^+^ (5 mM) and Tris–HCl buffer (100 mM, pH 6.5–9.0) or Gly-NaOH buffer (100 mM, pH 9.0–11.0). These were set up to investigate the pH dependence of the initial rates of *L*-glutamate, *rac*-Glu-γ-P_H_ or *L*-Glu-γ-P_H_ conversion by bovine GDH. Reactions were initiated by the addition of 1 µL (14 μg, 0.5 U, according to the definition given in Section 2.1) GDH in a 50% glycerol solution and carried out at 25 °C.

The kinetic parameters (*K*_m_ and *k*_cat_) for each tested compound were determined using reaction mixtures (500 μL) containing *L*-glutamate (0.1–10 mM) or *L*-Glu-γ-P_H_ (1–20 mM) in the presence of 5 mM NAD^+^ and 100 mM Tris–HCl buffer at pH 8.5. For high concentrations of *L*-Glu-γ-P_H_ (40–200 mM), a stock solution of *L*-Glu-γ-P_H_ in water (1.0 M) at pH 8.5 (pH was adjusted with aq. NaOH) was used. In these cases, 100 mM Tris–HCl buffer at pH 8.5 was also present in the substrate mixtures. Reactions were initiated by the addition of 1 µL (14 μg, 0.5 U, according to the definition given in Section 2.1) GDH in a 50% glycerol solution and carried out at 25 °C.

The reaction rates were quantified by monitoring the increase in NADH concentration using the molar absorption coefficient ε_340_ = 6220 M^−1^cm^−1^. The measurements were carried out continuously for the first 3 to 10 min, and the initial rates were fitted by linear regression of the kinetic curve. The dependence of the reaction rate on the substrate concentration was fitted to the standard Michaelis–Menten equation. Data processing was performed using Origin 2015.

### 2.3. The Extent of L-Glu or L-Glu-γ-P_H_ Conversion into α-KG or α-KG-γ-P_H_, Respectively

Reaction mixtures containing either 10 mM *L*-glutamate or 10 mM *L*-Glu-γ-P_H_ and 5 mM NAD^+^ at pH 9.0 (without buffer) were used to determine the extent of the conversion of *L*-glutamate and *L*-Glu-γ-P_H_ to α-KG and α-KG-γ-P_H_, respectively. Similar reaction mixtures in Tris–HCl buffer (100 mM, pH 9.0) were used as a control. Reactions were initiated by the addition of 1 µL (14 μg, 0.5 U, according to the definition given in Section 2.1) GDH in a 50% glycerol solution and carried out at 25 °C. The extent of substrate conversion was determined at the time points of 10 and 20 min and 1, 2, 3.5, 5, 7 and 24 h after the addition of the enzyme and by monitoring the increase in NADH concentration at 340 nm in the absorption spectrum range of 200–500 nm.

### 2.4. Preparative Synthesis of α-KG-γ-P_H_

To a mixture containing *L*-Glu-γ-P_H_ (167 mg, 1 mmol) and NAD^+^ (331 mg, 0.5 mmol) in water (100 mL), a NaOH solution at pH 9.0 was added, followed by the addition of 200 µL GDH (5.0 mg, 105 U, according to the definition given in Section 2.1) in a 50% glycerol solution. The accumulation of α-KG-γ-P_H_ was indirectly estimated by the increase in A_340_ at 10 and 20 min and 1, 2, 3.5, 5, 7 and 24 h after the start of the reaction. After 24 h, the reaction mixture was concentrated *in vacuo*. The residue was dissolved in water (2 mL), applied on the column packed with a Dowex 50 W X8 resin (H^+^ form, V = 40 mL), and eluted with water. Acidic fractions (1.0 mL each) eluted after the column-free volume were analyzed by ^31^P-NMR. Fractions containing α-KG-γ-P_H_ were concentrated in vacuo and dried over P_2_O_5_/KOH *in vacuo* to give 60 mg of a mixture containing α-KG-γ-P_H_ and NAD^+^ in a molar ratio of 1:2. The residue was diluted in 900 µL of deionized water and divided into 300 µL aliquots. Each aliquot was applied on a semi-preparative reverse-phase column 250 × 10 mm ReproSil-Pur C18-AQ (5 µm, Dr. Maisch GmbH, Ammerbuch, Germany), and HPLC purification was carried out using an isocratic system at a flow rate of 2 mL/min and column temperature of 25 °C, with solvent A, 0.2% aq. trifluoroacetic acid (TFA), as a mobile phase. Starting from 2 min, twenty 1 mL fractions were collected, dried *in vacuo*, dissolved in D_2_O, and analyzed by ^31^P-NMR. The HPLC column was regenerated by applying a linear washing gradient. Solvent A was 0.2% aq. TFA. Solvent B was 0.2% TFA in acetonitrile. The gradient profile was 0 to 100% B within 20 min at a flow rate of 2 mL/min, followed by column equilibration by passing solvent A for 10 min at the same flow rate.

The fractions containing pure α-KG-γ-P_H_ were concentrated *in vacuo*. The residue was co-evaporated *in vacuo* with water (3 × 2 mL) and dried over P_2_O_5_/KOH *in vacuo* to afford α-KG-γ-P_H_ (5 mg, 30% as calculated from the degree of *L*-Glu-γ-P_H_ conversion and 3% as calculated for starting *L*-Glu-γ-P_H_) as a semisolid oil. α-KG-γ-P_H_ (keto form) ^1^H NMR (300.13 MHz, H_2_O/D_2_O mixture) δ: 7.08 (dt, 1H, ^1^*J*_HP_ 541.8 Hz, ^3^*J*_HH_ 2.0 Hz, P-H), 3.12 (dt, 2H, ^3^*J*_HP_ 14.0 Hz, ^3^*J*_HH_ 7.5 Hz, -CH_2_-C=O), 2.10–1.90 (m, 2H, -CH_2_P). α-KG-γ-P_H_ (hydrated form) ^1^H NMR (300.13 MHz, H_2_O/D_2_O mixture) δ: 7.03 (dt, 1H, ^1^*J*_HP_ 538.8 Hz, ^3^*J*_HH_ 1.8 Hz, P-H), 2.10–1.90 (m, 2H, -CH_2_C(OH)_2_), 1.82–1.67 (m, 2H, -CH_2_P). α-KG-γ-P_H_ (keto form) ^13^C NMR (75.43 MHz, H_2_O/D_2_O mixture) δ: 198.1 (d, ^3^*J*_CP_ 12.5 Hz, >C=O), 164.5 (s, -COOH), 31.1 (d, ^2^*J*_CP_ 1.7 Hz, -CH_2_-C=O), 23.4 (d, ^1^*J*_CP_ 91.8 Hz, -CH_2_-P). α-KG-γ-P_H_ (hydrated form) ^13^C NMR (75.43 MHz, H_2_O/D_2_O mixture) δ: 174.3 (s, -COOH), 94.3 (d, ^3^*J*_CP_ 18.0 Hz, >C(OH)_2_), 30.2 (d, ^2^*J*_CP_ 1.5 Hz, -CH_2_-C(OH)_2_), 24.2 (d, ^1^*J*_CP_ 90.9 Hz, -CH_2_-P). α-KG-γ-P_H_ (keto form) ^31^P NMR (121.44 MHz, H_2_O/D_2_O mixture) δ: 32.7 (s). α-KG-γ-P_H_ (hydrated form) ^31^P NMR (121.44 MHz, H_2_O/D_2_O mixture) δ: 33.5 (s). The NMR spectra of α-KG-γ-P_H_ (keto and hydrated forms) are shown in Figures S3–S5.

## 3. Results

### 3.1. L-Glu-γ-P_H_ But Not D-Glu-γ-P_H_ Is a Substrate of GDH

**Figure 2:**
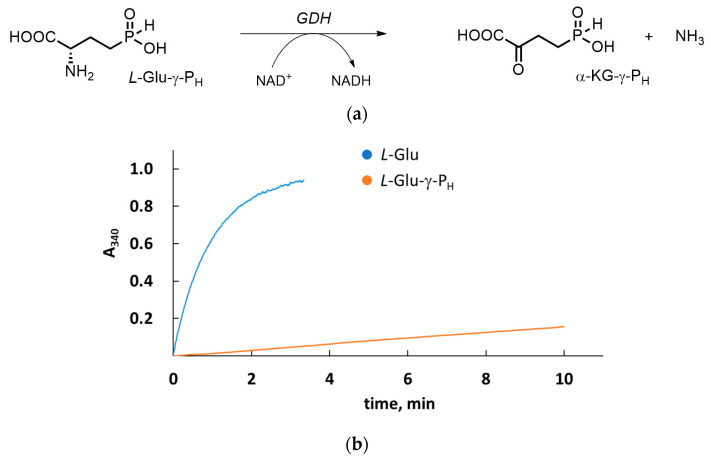
*L*-Glu-γ-P_H_ is a low-affinity substrate of GDH. (**a**) Schematic representation of the oxidative deamination of *L*-Glu-γ-P_H_ catalyzed by GDH, yielding α-KG-γ-P_H_. (**b**) Reactions (500 µL) were performed in Tris–HCl buffer (100 mM, pH 7.5) at 25 °C containing *L*-glutamate (10 mM) or *L*-Glu-γ-P_H_ (10 mM) and NAD^+^ (5 mM) and initiated by addition of GDH (14 µg). Data are from a representative experiment.

The substrate properties of *L*-Glu-γ-P_H_ (Figure 1b) on bovine liver GDH were initially investigated in Tris–HCl buffer at pH 7.5 at 25 °C (according to the instructions of the manufacturer details in Section 2.1) and compared with that of *L*-glutamate. Under these experimental conditions, the physiological substrate, *L*-glutamate, is rapidly (i.e., within 2 min) oxidized, and NADH is formed, as can be directly recorded by monitoring the increase in absorbance at 340 nm (the NADH absorbance maximum). Under the same experimental conditions, *L*-Glu-γ-P_H_ (10 mM) is oxidized at a much slower rate (Figure 2). It is worth remarking that 10 mM *L*-Glu-γ-P_H_ may be lower than the *K*_m_ value if taking, as reference, the known *K*_m_ value of *L*-Glu-γ-P_H_ in a PLP-dependent glutamate decarboxylase reaction and the *K*_m_ values of the α-amino-*H*-phosphinic analogues of methionine and tyrosine in methionine-γ-lyase and tyrosine phenol-lyase reactions ([17] and ref. within). Nevertheless, the substrate properties of *L*-Glu-γ-P_H_ on GDH can be determined a priori without exposing the enzyme to a disproportionally high *L*-Glu-γ-P_H_ concentration for the qualitative purpose of this preliminary investigation.

**Figure 3:**
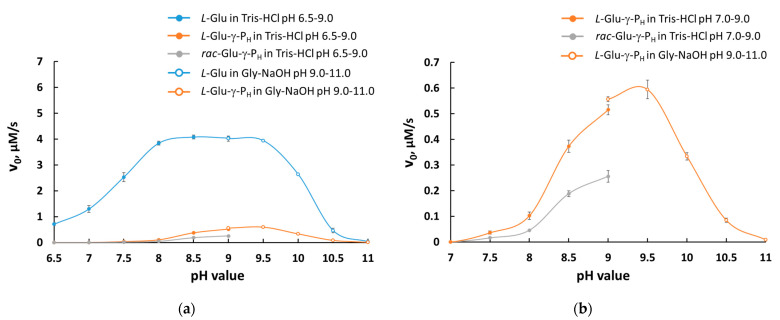
pH dependence of the initial reaction rates of GDH reaction with *L*-glutamate, *rac*-*L*-Glu-γ-P_H_ and *L*-Glu-γ-P_H_ as substrates. (**a**) Comparison of *L*-glutamate with *rac*-*L*-Glu-γ-P_H_ and *L*-Glu-γ-P_H_ as GDH substrates at different pH values. Reactions (500 µL) were performed in Tris–HCl buffer (100 mM, pH 6.5–9.0) and Gly-NaOH buffer (100 mM, pH 9.0–11.0) at 25 °C containing *L*-glutamate (10 mM), *rac*-Glu-γ-P_H_ (10 mM) or *L*-Glu-γ-P_H_ (10 mM) and NAD^+^ (5 mM) and initiated by addition of GDH (14 µg). (**b**) pH dependence of the GDH reaction rate for *rac*-*L*-Glu-γ-P_H_ and *L*-Glu-γ-P_H_ (in detail). Results are means ± SD of *n* = 3 independent assays, representative of *n* = 3 independent experiments.

To investigate in more detail the substrate properties of *L*-Glu-γ-P_H_, the pH dependence of the GDH reaction was studied in the pH range of 6.5–11.0. The reaction turned out to be faster in the pH range of 8–9 compared with the reaction rate at pH 7.5 (Figure 3). This turned out to be true for all tested compounds, i.e., *L*-glutamate, *L*-Glu-γ-P_H_ and *rac*-Glu-γ-P_H_. Again, in these experiments, both *L*-glutamate and *L*-Glu-γ-P_H_ were used at 10 mM, respectively. 

*L*-Glu-γ-P_H_ concentrations (1–200 mM) were used to determine the kinetic parameters of the reaction because amino-*H*-phosphinic analogues of amino acids, as a rule, have much higher *K*_m_ values compared with natural amino acids ([17] and ref. within). The *K*_m_ and *k*_cat_ values for *L*-Glu-γ-P_H_ at pH 8.5 were 52 ± 4 mM and 0.032 ± 0.002 s^−1^, respectively (Table 1, Figure S1). The catalytic efficiency *k*_cat_/*K*_m_ clearly indicates that, at pH 8.5, *L*-glutamate was 116 times a better substrate of bovine liver GDH compared to *L*-Glu-γ-P_H_ (Table 1).

When *D*-Glu-γ-P_H_ (20 mM) was used instead, under identical experimental conditions as above, no increase in absorbance at 340 nm was observed within 15 min at 25 °C, demonstrating the *D*-isomer of Glu-γ-P_H_ is not a substrate. This is in agreement with the halved activity of *rac*-Glu-γ-P_H_ (Figure 2) compared to *L*-Glu-γ-P_H_ at the same concentration (Figure 3). When 20 mM of *D*-Glu-γ-P_H_ was added to the GDH reaction mixture containing 1 mM *L*-glutamate, no decrease in the reaction rate was observed; hence, *D*-Glu-γ-P_H_ was presumed to be neither a substrate nor an inhibitor of GDH.

Thus, for the first time, it was demonstrated that *L*-Glu-γ-P_H_ is a substrate of bovine type II GDH, and the pH optimum of GDH reaction in the presence of this substrate does not reach its maximum at pH 8.5 as for *L*-glutamate [25,26], but it achieves this at pH 9.5 in Tris–HCl buffer. Above pH 9.0, in the Gly-NaOH buffer that was used to assay the activity for more alkaline pH (9.0–11.0), pH 9.5 was found optimal when *L*-Glu-γ-P_H_ was the substrate of GDH. Any other buffer system in the alkaline pH range tested (borate and carbonate buffers) supported the activity of the enzyme much worse than the Gly-NaOH buffer.

### 3.2. Glu-γ-P_5_ and PT Are Neither Substrates Nor Inhibitors of GDH

It is known that *D,L*-Glu-γ-P_5_, *rac*-Glu-γ-P_H_ and *D,L*-PT were 3–4 times less efficient as substrates of *E. coli* GABA-transaminase compared to GABA [27]. On the other hand, *D,L*-Glu-γ-P_5_ is neither a substrate nor an inhibitor of *E. coli* glutamate decarboxylase [17,28] and porcine heart aspartate aminotransferase [17]. As the binding of *H*-phosphinic, H_3_C-phosphinic and phosphonic glutamate analogues to each enzyme may be strongly affected by the enzyme active site conformation and the changes that it undergoes during catalysis, we carried out a deeper investigation on the substrate properties of *D,L*-Glu-γ-P_5_ and *D,L*-PT in the GDH reaction. Experiments were carried out in the pH range of 4.5–9.0 (Na–acetate buffer for pH 4.5–5.5, Na–phosphate buffer for pH 5.5–6.5 and Tris–HCl buffer for pH 6.5–9, using 1–40 mM concentrations of either *D,L*-Glu-γ-P_5_ or *D,L*-PT. Experiments were performed, as described above, for *L*-Glu-γ-P_H_. The assay was conducted in a wide pH range based on the existing data on biotechnological GDH-catalyzed *L*-PT synthesis from the corresponding α-ketophosphonate, i.e., 2-oxo-4-[(hydroxy)(methyl)phosphinoyl]butyric acid (PPO), where the pH optimum turned out to be pH 7.5 [29]. However, in the case of *D,L*-Glu-γ-P_5_ or *D,L*-PT, no increase in A_340_ was observed in the studied pH range (4.5–9.0), indicating the absence of the substrate properties of *D,L*-Glu-γ-P_5_ and *D,L*-PT in the GDH reaction. Moreover, the inhibitory properties of Glu-γ-P_5_ and PT were studied, as described above, for *D*-Glu-γ-P_H_, and neither *D,L*-Glu-γ-P_5_ nor *D,L*-PT were found to be GDH inhibitors.

These data indicate that the replacement of the *H*-phosphinic group with a bulkier phosphonic or methylphosphinic group leads to the loss of the affinity of Glu-γ-P_5_ and PT to the enzyme. Very likely, the enzyme binding site of the distal carboxylate (i.e., in the γ-position) of *L*-glutamate can accommodate the *H*-phosphinic group of *L*-Glu-γ-P_H_, which is a bioisostere of the carboxylic group of *L*-glutamate. In the case of *D,L*-Glu-γ-P_5_ and *D,L*-PT, steric hindrance and the additional negative charge (in the case of *D,L*-Glu-γ-P_5_) make it incompatible with functional binding.

### 3.3. Comparison of the Percentage of Conversion of the Substrates Involved in GDH Reaction

**Figure 4:**
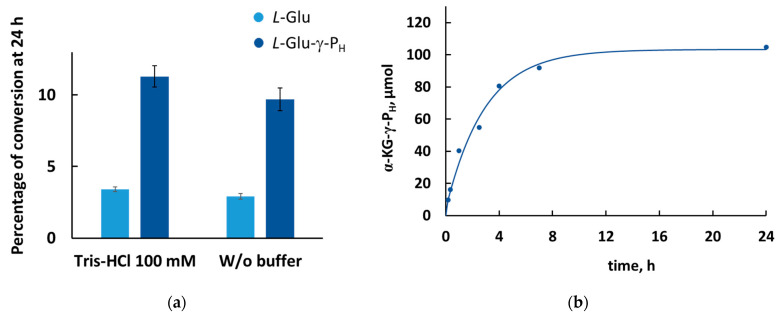
Enzymatic synthesis of α-KG-γ-P_H_ from *L*-Glu-γ-P_H_ using GDH. (**a**) The percentage of *L*-glutamate and *L*-Glu-γ-P_H_ conversion into α-KG and α-KG-γ-P_H_, respectively, with/without buffer. Reactions (500 µL) were performed without buffer at pH 9.0 in a mixture containing *L*-glutamate (10 mM) or *L*-Glu-γ-P_H_ (10 mM) and NAD^+^ (5 mM) at 25 °C, initiated by the addition of GDH (14 µg). A similar mixture in Tris–HCl buffer (100 mM, pH 9.0) was used as a control. Results are shown as means ± SD of *n* = 3 independent assays, representative of *n* = 3 independent experiments. (**b**) Accumulation of α-KG-γ-P_H_ over time in preparative synthesis. Reaction (100 mL) was performed at 25 °C in a buffer-free system at pH 9.0 in a mixture containing *L*-Glu-γ-P_H_ (10 mM) and NAD^+^ (5 mM) and initiated by addition of GDH (5 mg).

The above results demonstrate that *L*-Glu-γ-P_H_ is a substrate of GDH and that an enzymatic synthesis of α-KG-γ-P_H_ is possible. The extent of conversion of *L*-Glu-γ-P_H_ (10 mM) to α-KG-γ-P_H_ reached 11% (Figure 4a) after 24 h when the reaction mixture (500 µL) at 25 °C contained NAD^+^ (5 mM) and GDH (14 µg) in Tris–HCl buffer (100 mM, pH 9.0). 

Surprisingly, the percentage of conversion of *L*-Glu-γ-P_H_ was about three times higher than that of *L*-glutamate under the same reaction conditions (Figure 4a). This may be attributed to the weaker affinity of α-KG-γ-P_H_ to the enzyme compared with α-KG, and this is consistent with the low affinity of *L*-Glu-γ-P_H_, with a *K*_m_ value that is 58 times higher than that of *L*-glutamate (Table 1). Thereby, the stability of the abortive complex GDH∙NAD^+^∙α-KG-γ-P_H_, formed during the enzymatic reaction, may prevent the conversion of *L*-Glu-γ-P_H_ to α-KG-γ-P_H_ less effectively than the complex GDH∙NAD^+^∙α-KG when *L*-glutamate is used as a substrate.

### 3.4. Enzymatic Synthesis of H-Phosphinic Analogue of α-Ketoglutarate

With the aim of setting up a preparative enzymatic synthesis of α-KG-γ-P_H_, the reaction was performed in a buffer-free system at pH 9.0. Under these conditions, the pH dropped to 8.7 after 24 h, and the percentage of conversion of *L*-Glu-γ-P_H_ into α-KG-γ-P_H_ was about 9%, i.e., slightly decreased compared to the same reaction carried out in Tris–HCl buffer (Figure 4a). 

Preparative synthesis of α-KG-γ-P_H_ was carried out by scaling up the reaction volume to 100 mL using *L*-Glu-γ-P_H_ (10 mM) and NAD^+^ (5 mM). The 2:1 ratio of *L*-Glu-γ-P_H_:NAD^+^ provided a reasonable balance between the conversion degree of *L*-Glu-γ-P_H_ into α-KG-γ-P_H_ and the ease of product isolation. The use of such a diluted solution was also necessary because NAD^+^ in high concentration inhibits GDH [30], as observed at 20 mM NAD^+^ in the substrate mixture (Figure S2). The percentage of the conversion of *L*-Glu-γ-P_H_ to α-KG-γ-P_H_ reached a plateau within 12 h (Figure 4b); the overall conversion was about 10%, and the α-KG-γ-P_H_/NAD^+^ ratio in the substrate mixture was 1:4 (1 mM:4 mM). Notably, further addition of fresh GDH did not restart the reaction. Ion-exchange chromatography on a Dowex 50W-X8 (H^+^ form) was used with water as an eluent to separate the target α-KG-γ-P_H_ from the unreacted *L*-Glu-γ-P_H_ and most of NAD^+^ and NADH. However, the eluted product still contained the coenzyme, i.e., the α-KG-γ-P_H_/NAD^+^ ratio was 1:2. Final purification by semi-preparative RP-HPLC yielded 5 mg of pure α-KG-γ-P_H_. The yield of the reaction was 3%, as calculated from the initial *L*-Glu-γ-P_H_ amount, given that the percentage of conversion of *L*-Glu-γ-P_H_ was only about 9% (Figure 4a). 

### 3.5. NMR Analysis of the Structure of α-KG-γ-P_H_

**Figure 5:**
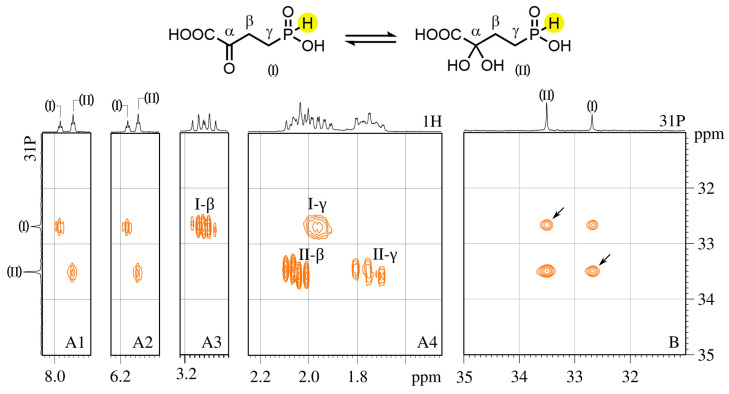
NMR spectra of α-KG-γ-P_H_ existing in aqueous solution as an equilibrium mixture of keto (I) and dihydroxy (II) forms (equation on the top). Panels (**A1**–**A4**): ^1^H-^31^P HMBC spectrum cut-away views containing ^31^P correlation cross-peaks with phosphorus-bound proton highlighted yellow in formulas above. Panel (**B**): ^1^H-decoupled ^31^P-^31^P NOESY spectrum displaying strong (I) and (II) exchange peaks (pointed by arrow marks). Note: vertical scale is common for all panels.

Enzymatically synthesized α-KG-γ-P_H_ in water (pH 1.35) exists as an equilibrium mixture of keto (I) and geminal diol (II) forms, and two signals at 33.5 and 32.7 ppm are detected in the ^31^P NMR spectrum, respectively (vertical projection on the left of Figure 5 and Figure S5). Forms (I) and (II) are interconverting at a slow rate *τ*^−1^ < 10 s^−1^ (estimated from well-resolved phosphorus-bound proton signals), giving rise to two separate sets of NMR signals in ^1^H, ^31^P and ^13^C spectra (Figures S3–S5). Strong exchange cross-peaks observed in the ^31^P-^31^P NOESY spectrum (Figure 5B) confirm that these duplicate signals are indeed arising from the same compound in the equilibrium between the two forms (Figure 5). Well-resolved phosphorus-bound proton signals in the ^1^H spectrum appear as two pairs of triplets near 7.95 and 6.15 ppm (Figure 5(A1,A2); see Figure S3 for the full spectrum). The low-field signal within each pair belongs to the keto form (I). Splitting between the pairs of triplets is about 540 Hz, which is characteristic of direct (single-bond) ^1^H-^31^P coupling. The equilibrium between the keto form (I) and geminal diol form (II) depends on the pH value. This dependence is revealed by different signal intensities in the ^31^P spectra recorded at pH 1.35 and 7.0 (Figure S5). Form (II) is dominating at acidic pH values (Figure S5A), whereas about 90% of the keto form (I) is present at pH 7.0, where signals of both forms experience an up-field shift of about 4.5 ppm (Figure S5). The assignment of signals pertaining to forms (I) and (II) follows clearly from the ^13^C NMR spectrum, where the characteristic α-carbonyl signal at 198.1 ppm is indicative of the keto form (I), and all other signals of the keto form (I) and geminal diol form (II) are also well resolved (Figure S4). Interestingly, the ^31^P-^13^C coupling constant for β-carbon in the keto form (I) is smaller than that for α-carbon at 1.7 and 12.5 Hz, respectively (Figure S4); the same is true for the geminal diol form (II). The ^1^H-^31^P HMBC spectrum allowed us to unequivocally assign all the signals of the keto (I) and geminal diol (II) forms, including partly overlapping signals of the γ-CH_2_ group of (I) and the β-CH_2_-group of (II) (Figure 5(A4)). 

## 4. Discussion

**Figure 6:**
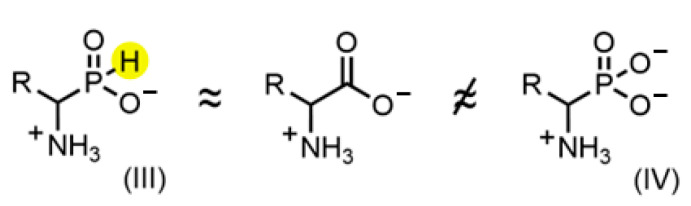
Chemical structures of α-amino-*H*-phosphinic (III) and α-aminophosphonic acids (IV) confronted with amino acids (in the middle). The *H*-phosphinic group possesses a hydrogen atom (highlighted in yellow) that allows this group to acquire a flattened tetrahedral geometry making it bioisostere of the carboxyl group.

The substitution of the carboxylic group of amino acids with an acidic phosphorus-containing moiety results in two analogue classes, α-amino-*H*-phosphinic (III) and α-aminophosphonic acids (IV), depicted in Figure 6. The *H*-phosphinic group with a charge of “-1” assumes a flattened tetrahedron geometry (the size of the hydrogen atom is much smaller than that of the hydroxyl group of aminophosphonates). Therefore, it is a bioisostere of the planar single-charged carboxylic group [17]. Respectively, *H*-phosphinic analogues (III) have been shown to be the substrates of the enzymes of amino acid metabolism, such as PLP-dependent alanine aminotransferase [31], aspartate aminotransferase, methionine-γ-lyase and tyrosine phenol-lyase ([17] and ref. within). 

Substitution of the distal carboxylic group of *L*-glutamate gives rise to *L*-Glu-γ-P_H_ (Figure 1b and Figure 5), which was demonstrated to be a substrate of GABA transaminase [27], aspartate aminotransferase and glutamate decarboxylase ([17] and ref. within). In the latter case, the kinetic resolution of *rac*-Glu-γ-P_H_ was exploited to obtain preparative amounts of GABA-P_H_ and *D*-Glu-γ-P_H_ [17]. The former was enzymatically transaminated into the *H*-phosphinic analogue of succinic acid semialdehyde, which was oxidized into the *H*-phosphinic analogue of succinate by NAD^+^-dependent succinic semialdehyde dehydrogenase [17]. Such a transformation is non-trivial since it is known that phosphorous acid (H_3_PO_3_), also having *P–H* bonds, is efficiently oxidized to phosphoric acid (H_3_PO_4_) by NAD^+^-dependent dehydrogenase [32]. However, the *H*-phosphinic analogue of succinic acid semialdehyde was instead oxidized to the corresponding succinate analogue without affecting the *P-H* bonds [17]. These enzymatic data are of importance as they demonstrate that, once they penetrate the cell, *H*-phosphinic analogues of amino acids on distal carboxyl groups can undergo substrate-like transformations, providing new compounds with *C-P-H* bonds, some of which may have biochemical targets that are different from that of the parent amino acid analogue.

GDH catalyzes the reversible oxidative deamination of *L*-glutamate to α-ketoglutarate. This is one of the most important metabolic pathways affecting nitrogen metabolism, the Krebs cycle and the acid base and redox balance of the cell [20,33]. Nonetheless, the ability of *L*-Glu-γ-P_H_ to undergo NAD^+^-dependent oxidative deamination to form α-KG-γ-P_H_ has not been studied yet. Hence, here, we have investigated the interaction of *L*-Glu-γ-P_H_ with GDH in comparison with that of glutamate phosphonic analogues, i.e., *D,L*-Glu-γ-P_5_ and *D,L*-PT (Figure 1a,b). *L*-Glu-γ-P_H_ containing reactive *P-H* bond distanced from the reaction center turned out to be a GDH substrate despite the oxidative nature of NAD^+^. The pH optimum of the NAD^+^-dependent oxidative deamination of *L*-Glu-γ-P_H_ was shifted to a slightly alkaline region (Figure 2), and the *K*_m_ value was 58 times higher than that of *L*-glutamate, while *k*_cat_ exhibited only a twofold decrease (Table 1). Although the *H*-phosphinic group is a bioisostere of the carboxylic group, its flattened tetrahedral geometry still affected the binding of *L*-Glu-γ-P_H_ to the active site of GDH.

We also found that *D,L*-Glu-γ-P_5_ and *D,L*-PT, with bulky phosphorus-containing groups, did not exhibit substrate properties in the GDH reaction within a wide pH range. This is in line with the known ability of aminophosphonic acids (IV, Figure 6) and their derivatives to model the tetrahedral transition state of the carboxylic group [34] but not the substrate (distal) carboxylic group ([17] and ref. within [35]). However, the enzymes GABA aminotransferase [27] and Dnmt3a [36] are exceptions. In both cases, the substrate properties of the corresponding *H*-phosphinic and phosphonic analogues were close. A notable example of the substrate properties of an amino acid phosphorus-containing analogue with a bulky substituent is the enzymatic synthesis of *L*-PT from a corresponding α-ketoglutarate analogue catalyzed by the *Pseudomonas putida* GDH mutant [29,37]. However, this was not the case with the NAD^+^-dependent oxidative deamination of *D,L*-Glu-γ-P_5_ and *D,L*-PT catalyzed by type II bovine liver GDH, and neither *D,L*-Glu-γ-P_5_ nor *D,L*-PT were the substrates of this enzyme in the wide pH range assayed in this study.

The equilibrium of the reversible GDH reaction favors the conversion of α-KG to *L*-glutamate [20]. As a consequence, the yield of α-KG synthesis from *L*-glutamate is low (Figure 4a). The same is true for the conversion of *L*-Glu-γ-P_H_ into α-KG-γ-P_H_ (Figure 4a). First, the enzymatic synthesis of α-KG-γ-P_H_, (Figure 4b) was carried out with a relatively high concentration of NAD^+^ due to the absence of a NADH-NAD^+^ regeneration system, which is used in the case of biotechnological applications of NAD^+^-involving reactions. NAD^+^ is known as a GDH coenzyme inhibitor [30], and the NAD^+^ concentration used in preparative synthesis was kept at 5 mM (Figure S2). This made it reasonable to use only 10 mM *L*-Glu-γ-P_H_ despite this concentration being much lower than the *K*_m_ value of *L*-Glu-γ-P_H_ (Table 1). Therefore, the possibility of the conversion of *L*-Glu-γ-P_H_ into α-KG-γ-P_H_ was demonstrated for the first time, though for its biotechnological implementation, coupling with NAD^+^ regeneration will be required. 

## 5. Conclusions

Substitution of the carboxylic group of amino acids with an acidic phosphorus group containing unusual hydrogen–phosphorus–carbon (*H-P-C*) bonds results in amino *H*-phosphinic acids exhibiting diverse biological activity. This may be attributed to the effects of either the amino *H*-phosphinic acid itself or its metabolites, which also contain *C-P-H* bonds. The biochemical targets of the *H*-phosphinic amino acids and the corresponding metabolites differ, ensuring a multitarget effect. NAD-dependent dehydrogenases are among the key enzymes linking the metabolism of amino and keto acids. In this work, we demonstrate for the first time that the distal *H*-phosphinic analogue of glutamate serves as a substrate for bovine liver glutamate dehydrogenase, and we performed the enzymatic synthesis of the distal *H*-phosphinic analogue of α-ketoglutarate, disclosing the peculiarities of the interaction of distal phosphorus-containing analogues of glutamic acid with glutamate dehydrogenase.

## 6. Patents

A patent IT 102016000098005 has been granted, which includes α-KG-γ-P_H_, an antibacterial agent, the enzymatic synthesis of which is described in detail in this study (https://www.uniroma1.it/en/brevetto/102016000098005; URL last accessed on 22 November 2024).

## Figures and Tables

**Table 1:** Kinetic parameters of *L*-glutamate and *L*-Glu-γ-P_H_ in GDH (14 µg) reaction at pH 8.5.

	*K*_m_, mM	*k*_cat_, s^−1^	*k*_cat_/*K*_m_, s^−1^∙M^−1^
*L*-Glu	0.9 ± 0.2 ^1^	0.065 ± 0.003	70 ± 20
*L*-Glu-γ-P_H_	52 ± 4	0.032 ± 0.002	0.6 ± 0.1

^1^ 1.62 mM at pH 7.5 [24].

## Data Availability

Additional data are available in the Supplementary Materials. If not present there, they can be requested from the authors upon a reasonable and motivated request.

## Supplementary Materials

The following supporting information can be downloaded at: https://www.mdpi.com/article/10.3390/biom14121574/s1, Figure S1: The initial rate of the GDH reaction depends on the concentrations of *L*-glutamate and *L*-Glu-γ-P_H_; Figure S2: High concentrations of NAD^+^ inhibit GDH reaction; Figure S3: ^1^H-NMR spectrum of α-KG-γ-P_H_ existing in aqueous solution as an equilibrium mixture of keto and dihydroxy forms, I and II; Figure S4: ^13^C-NMR spectrum of α-KG-γ-P_H_ existing in aqueous solution as an equilibrium mixture of keto and dihydroxy forms, I and II; Figure S5: ^31^P-NMR spectrum of α-KG-γ-P_H_ existing in aqueous solution as an equilibrium mixture of keto and dihydroxy forms, I and II.
