# Supplementary material for: Enzymatic Synthesis of Biologically Active H-Phosphinic Analogue of α-Ketoglutarate
Source: Biomolecules. 2024 Dec 10;14(12):1574. doi: 10.3390/biom14121574 (PMC11673680; doi:10.3390/biom14121574)
Supplement: Supplementary file 1 [file biomolecules-14-01574-s001.zip › biomolecules-3300725-supplementary.pdf]

Supplementary information

Enzymatic Synthesis of Biologically Active *H*-Phosphinic Analogue of *α*-Ketoglutarate

Vsevolod L. Filonov [1], Maxim A. Khomutov [1], Yaroslav V. Tkachev [1], Artem V. Udod [1], Dmitry V. Yanvarev [1], Fabio Giovannercole [2], Elena N. Khurs [1], Sergei N. Kochetkov [1], Daniela De Biase [3] and Alex R. Khomutov [1,*]

[1] Engelhardt Institute of Molecular Biology, Russian Academy of Sciences, Vavilov St., 32, 119991 Moscow, Russia

[2] Département de Biologie, Université de Namur, Rue de Bruxelles 61, 5000 Namur, Belgium

[3] Department of Medico-Surgical Sciences and Biotechnologies, Sapienza University of Rome, Corso della Repubblica 79, 04100 Latina, Italy

* Correspondence: alexkhom@list.ru

Contents:

Figure S1. The initial rate of the GDH reaction depends on the concentrations of *L*-glutamate and *L*-Glu-γ-P_H. S2

Figure S2. High concentrations of NAD^+ inhibit GDH reaction S2

Figure S3. ^{1}H-NMR spectrum of α-KG-γ-P_H existing in aqueous solution as an equilibrium mixture of keto- and dihydroxy forms, I and II. S3

Figure S4. ^{13}C-NMR spectrum of α-KG-γ-P_H existing in aqueous solution as an equilibrium mixture of keto- and dihydroxy forms, I and II. S4

Figure S5. ^{31}P-NMR spectrum of α-KG-γ-P_H existing in aqueous solution as an equilibrium mixture of keto- and dihydroxy forms, I and II. S5

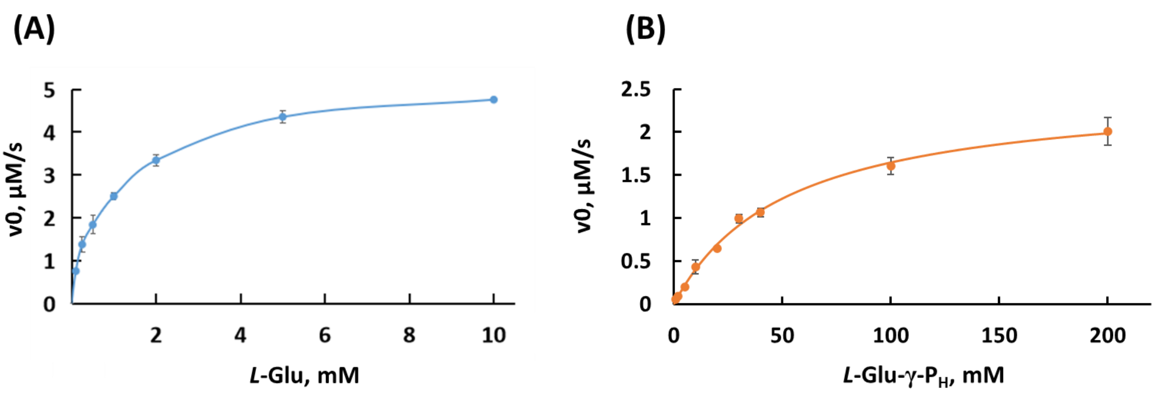

Figure S1. The initial rate of the GDH reaction is determined by the concentration of *L*-glutamate (A) and *L*-Glu-γ-P_H (B). Reactions (500 µL) were carried out in Tris-HCl buffer (100 mM, pH=8.5) at 25ºC containing *L*-glutamate (0.1-10 mM) or *L*-Glu-γ-P_H (1-200 mM), NAD^+ (5 mM). In the case of high concentrations of *L*-Glu-γ-P_H (40-200 mM) a stock solution of *L*-Glu-γ-P_H in water (1.0 M) adjusted to pH 8.5 with aq. NaOH was used. In these cases, 100 mM Tris-HCl buffer, pH 8.5 was also present in the substrate mixtures. Reactions were initiated by the addition of GDH (14 µg). Results are shown as means ± SD of n=3 independent assays, representative of n=3 independent experiments.

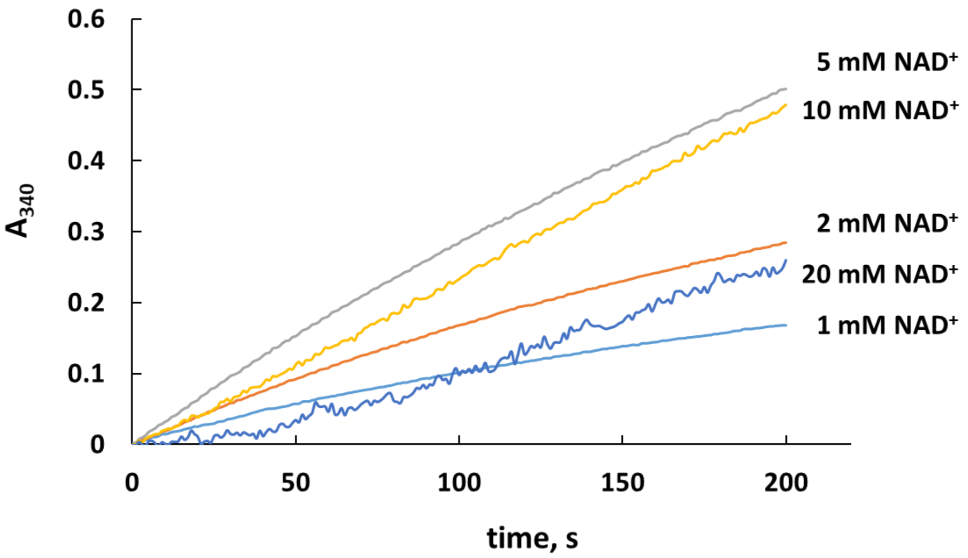

Figure S2. High concentrations of NAD^+ inhibit GDH reaction. Reactions (500 µL) were carried out in Tris-HCl buffer (100 mM, pH=9.0) at 25ºC containing *L*-Glu-γ-P_H (10 mM); NAD^+ (1, 2, 5, 10 or 20 mM) and initiated by the addition of GDH (14 µg).

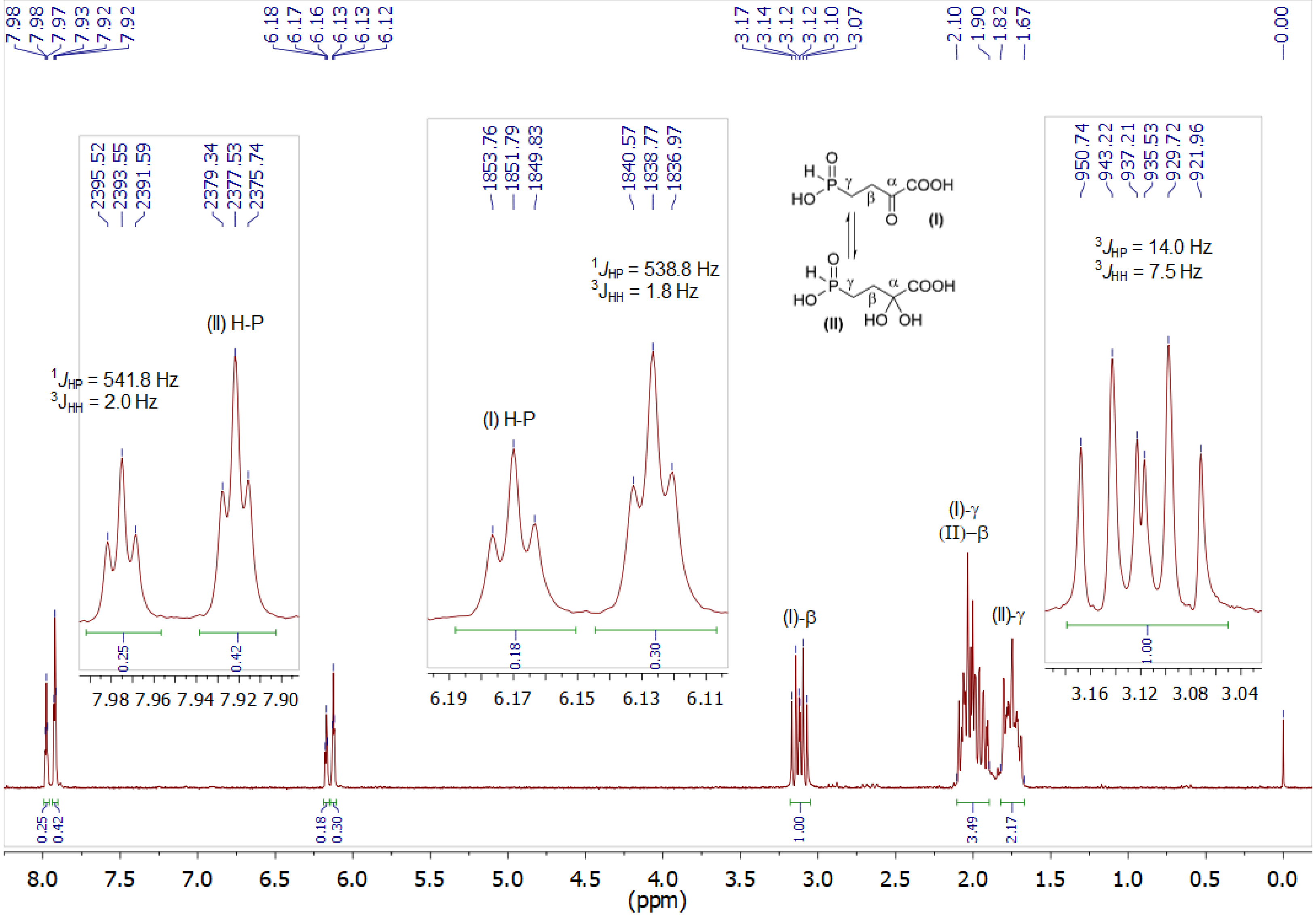

Figure S3. **^{1}H-NMR spectrum of α-KG-γ-P$_H$ existing in aqueous solution as an equilibrium mixture of keto- and dihydroxy forms, I and II.** Spectrum was recorded in H_2O/D_2O mixture 9/1 at pH 1.35. Water signal was suppressed using "excitation sculpting" pulse sequence.

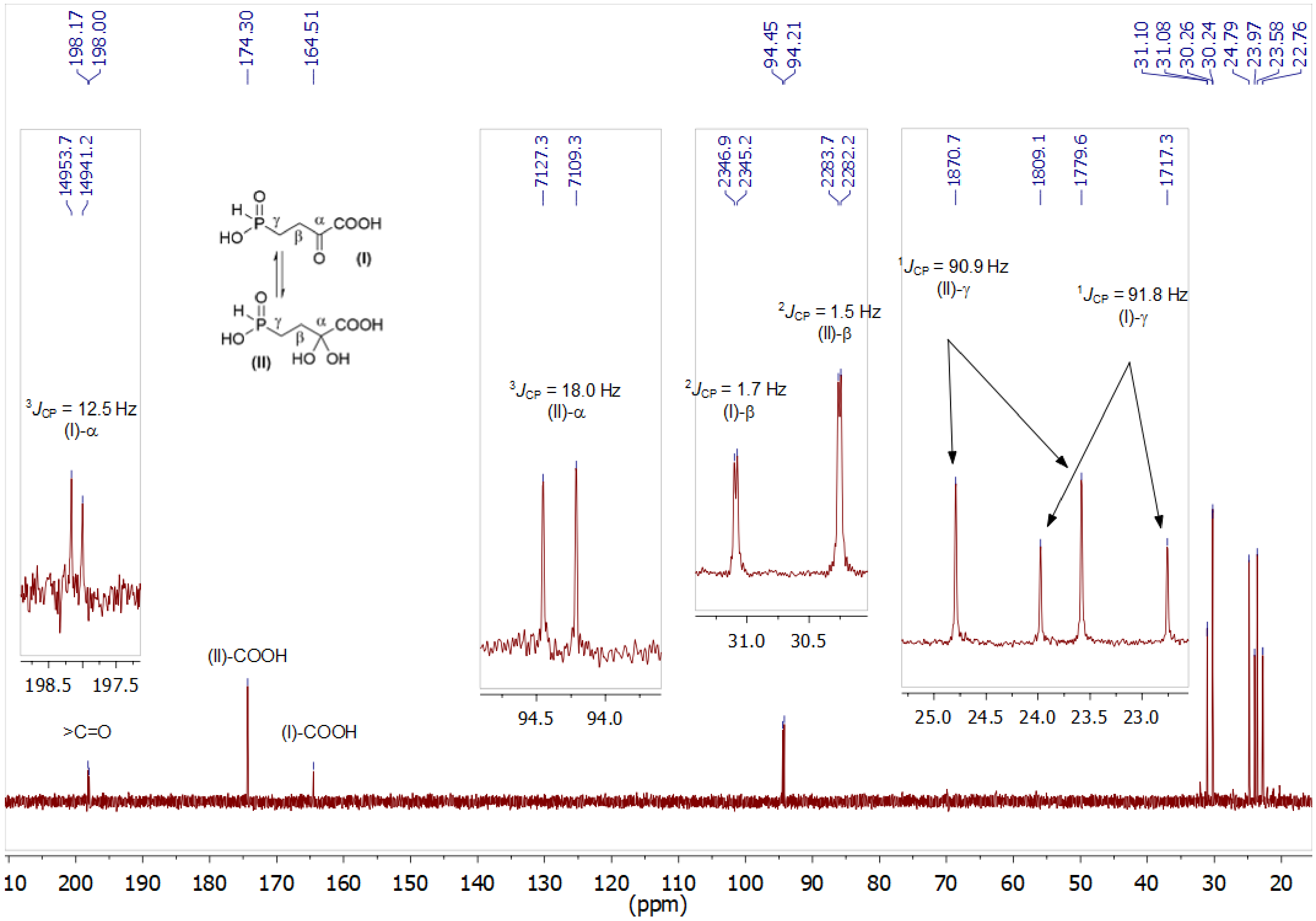

Figure S4. ^{13}**C-NMR spectrum of α-KG-γ-P$_H$ existing in aqueous solution as an equilibrium mixture of keto- and dihydroxy forms, I and II.** Spectrum was recorded at ph 1.35

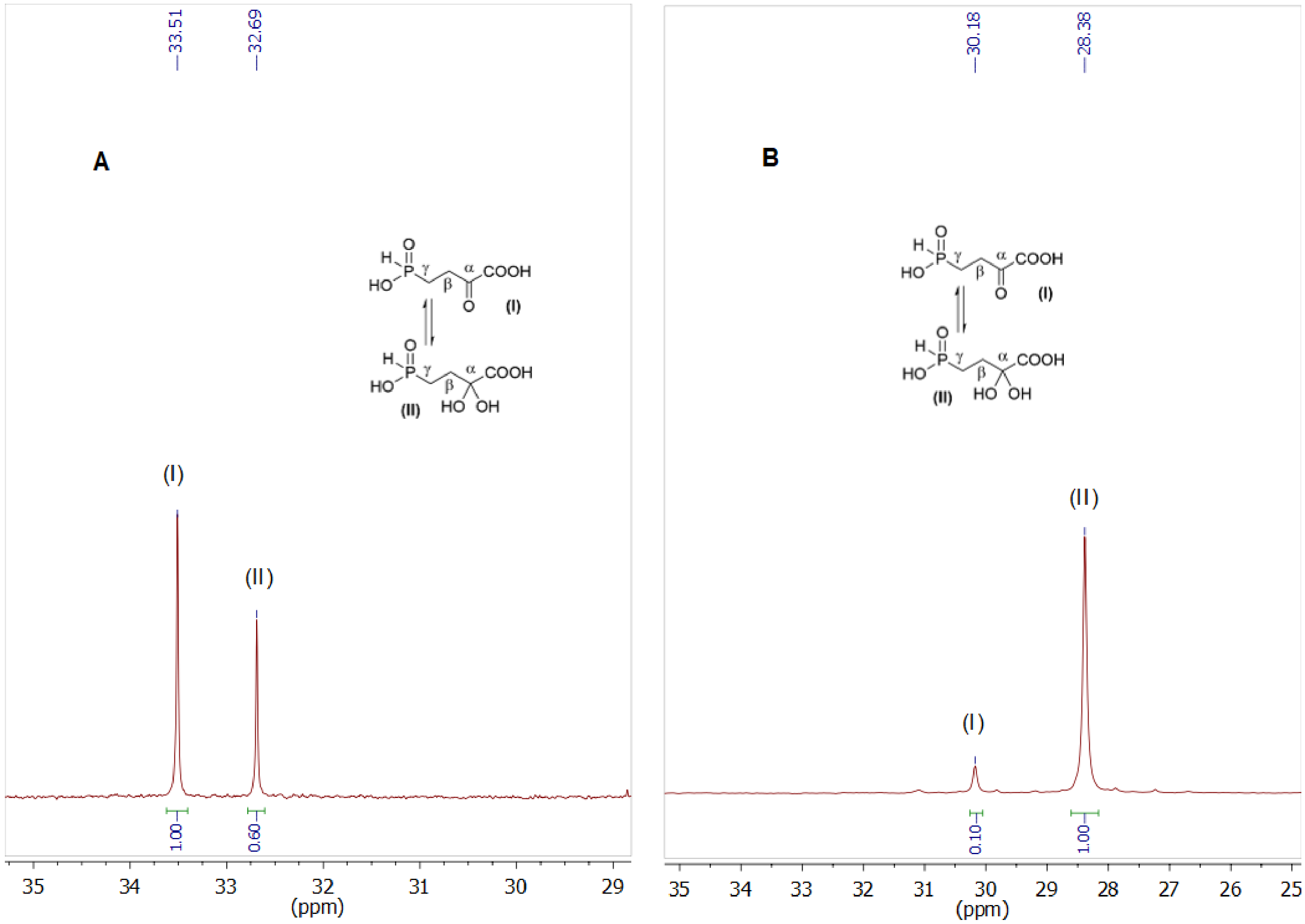

Figure S5. **^{31}P-NMR spectra of α-KG-γ-P_H in aqueous solution as an equilibrium mixture of keto (I) and geminal diol (II) forms.** Spectrum at pH 1.35 (A); spectrum at pH 7.0 (B).
